# Impact of Cardiovascular–Kidney–Metabolic Syndrome Staging on Myocardial Infarction Outcomes: A Retrospective Analysis of 2.7 Million Patients

**DOI:** 10.3390/diseases13040097

**Published:** 2025-03-27

**Authors:** Ronny Shabtai, Marlon Villaga Gatuz, Adam Folman, Maguli S. Barel, Rami Abu-Fanne, Dmitry Abramov, Mamas A. Mamas, Ariel Roguin, Ofer Kobo

**Affiliations:** 1 Adelson School of Medicine, Ariel University, Ariel 4070000, Israel; shabtai.ronny@gmail.com; 2 Department of Cardiology, Hillel Yaffe Medical Center, Hadera 3820302, Israel; mvg.cardio@gmail.com (M.V.G.); adamfolman@gmail.com (A.F.); maguilb@hymc.gov.il (M.S.B.); rabufanne@gmail.com (R.A.-F.); aroguin@technion.ac.il (A.R.); 3 Department of Cardiology, Linda Loma University Health, Linda Loma, CA 92354, USA; dabramov1@gmail.com; 4 Keele Cardiovascular Research Group, Keele University, Newcastle ST5 5BG, UK; mamasmamas1@yahoo.co.uk; 5 National Institute for Health and Care Research (NIHR) Birmingham Biomedical Research Centre, Birmingham B15 2TH, UK

**Keywords:** cardiovascular–kidney–metabolic syndrome, acute myocardial infarction, risk factors, outcomes

## Abstract

**Background**: Cardiovascular–kidney–metabolic (CKM) syndrome, recently defined by the American Heart Association, encompasses the interplay between obesity, diabetes, chronic kidney disease, and cardiovascular disease. This study aimed to investigate the impact of CKM syndrome severity on outcomes in patients with acute myocardial infarction (AMI). **Methods**: A retrospective analysis was conducted using the National Inpatient Sample database from 2016 to 2019. Adult patients hospitalized with AMI were stratified into CKM Stages 0–4 based on ICD-10 codes. Multivariable logistic regression models were used to examine associations between CKM stages and in-hospital procedures and outcomes. **Results**: The study analyzed 2,768,154 AMI cases. Advanced CKM stages were associated with older age and a higher proportion of males. Patients with severe CKM were more likely to undergo invasive procedures. Coronary angiography showed the strongest association in CKM Stage 4A (aOR: 6.86, 95% CI: 6.73–6.99, *p*-value < 0.001) and Stage 4B (aOR: 3.87, 95% CI: 3.80–3.95, *p*-value < 0.001). Similarly, the likelihood of PCI was highest in Stage 4A (aOR: 5.93, 95% CI: 5.79–6.08, *p*-value < 0.001) and Stage 4B (aOR: 4.14, 95% CI: 4.04–4.24, *p*-value < 0.001). Notably, patients with CKM Stage 0 demonstrated higher odds of adverse outcomes compared to other stages. **Conclusions**: This study reveals a complex relationship between CKM syndrome severity and AMI outcomes. Patients with advanced CKM stages were more likely to undergo invasive procedures, and those without CKM risk factors unexpectedly showed worse outcomes. Among Stages 1–4B, no consistently graded association emerged between the CKM stage and adverse outcomes. These findings warrant further investigation into underlying mechanisms and long-term prognosis.

## 1. Introduction

Cardiovascular diseases (CVDs) remain a leading cause of death worldwide. Among these, acute myocardial infarction (AMI) is a significant contributor to mortality, particularly in developed countries. AMI affects approximately 3 million people globally and causes over 1 million deaths annually in the United States alone [1].

Cardiovascular–kidney–metabolic (CKM) syndrome describes a medical condition resulting from pathophysiological interactions among obesity, diabetes, chronic kidney disease (CKD), and CVD [2]. Recently defined by the American Heart Association (AHA) in 2023, CKM syndrome is common in the general population [3] and is associated with an elevated risk of mortality and morbidity [2]. Nearly every organ in the body can be affected by CKM syndrome; however, its most significant impact on morbidity and clinical outcomes is observed in CVD [2]. The AHA has established a staging system for CKM syndrome, ranging from Stage 0 to Stage 4. Stage 0 represents individuals with no CKM risk factors, while Stage 4 is characterized by clinically significant cardiovascular disease in individuals with excess or dysfunctional adiposity, other metabolic risk factors, or CKD [2].

Although CKM syndrome is known to increase the risk of CVD, data on the management and outcomes among MI patients at different stages of CKM remain limited. This study aims to address this knowledge gap by examining the relationship between CKM syndrome severity and MI patient outcomes, focusing on its potential impact on in-hospital procedures and clinical endpoints.

## 2. Methods

### 2.1. Data Source

This study utilized the National Inpatient Sample (NIS), a comprehensive healthcare database that is part of the Healthcare Cost and Utilization Project (HCUP). Established in 1988, the NIS represents one of the most extensive publicly accessible all-payer inpatient datasets in the United States. It encompasses approximately 7 million hospital stays annually, constituting a 20% stratified sample of discharges from U.S. community hospitals while excluding rehabilitation and long-term acute care facilities. The NIS serves as a valuable resource for researchers and policymakers, offering insights into various aspects of healthcare delivery across the nation, including trends in utilization, access, charges, quality, and outcomes at local, regional, and national levels [4].

### 2.2. Study Design and Population

**Figure 1:**
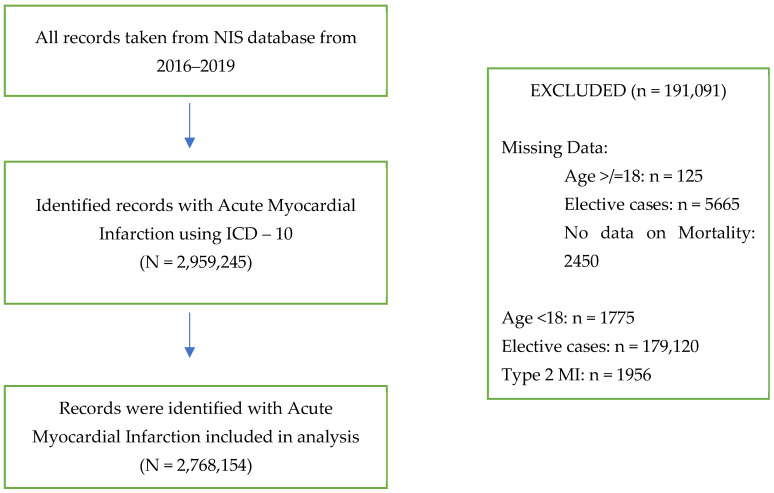
Flow diagram.

In this retrospective study, we conducted a comprehensive analysis of adult patients (aged ≥18 years) hospitalized between 2016 and 2019 with a diagnosis of acute myocardial infarction (AMI) (Figure 1). These patients were chosen based on the International Classification of Diseases, Tenth Revision, Clinical Modification (ICD-10-CM) diagnosis codes. Table S1 lists the ICD-10 codes used to define patient and procedural characteristics.

CKM syndrome is categorized by the American Heart Association (AHA) using a staging system (Table S2) ranging from Stage 0 to Stage 4 [2]. In this study (Table S3), the population was divided into five groups based on pre-existing CKM stage based on the data available from ICD-10 codes. Stage 0 refers to individuals without CKM risk factors, while Stage 1 includes early risk factors such as abdominal obesity and prediabetes. Due to challenges in distinguishing asymptomatic CVD in Stage 3, we combined Stages 2 and 3 into a single category. This combined stage contains metabolic conditions such as type 2 diabetes and chronic kidney disease, along with potential subclinical cardiovascular or kidney disease. Stage 4 is further divided into 4a (established cardiovascular disease without kidney failure) and 4b (with kidney failure).

Patient demographics were recorded for each hospital discharge, including age, gender, race, admission day (weekday or weekend), expected primary payer, and median household income according to ZIP code. Missing data on age, gender, elective and weekend admission, and mortality status were excluded from the analysis. Also, patients with type 2 MI documentation or elective admissions were excluded from the analysis. Each discharge record contained data on up to 30 diagnoses. ICD 10-CM codes were also used to classify complications and procedural information during hospitalization, including coronary angiography (CA), percutaneous coronary intervention (PCI), coronary artery bypass graft (CABG), thrombolysis, and use of mechanical ventilation and circulatory support.

### 2.3. Outcomes

The primary outcomes of this study were major adverse cardiovascular and cerebrovascular events (MACCEs) and all-cause mortality. MACCEs encompassed all-cause mortality, acute ischemic stroke or transient ischemic attack, and cardiac complications, including coronary artery dissection, pericardial effusion, Dressler’s syndrome, post-MI angina, intracardiac thrombus, and acute mechanical complications. Secondary outcomes comprised acute ischemic stroke, major bleeding events (defined as gastrointestinal, retroperitoneal, intracranial, or intracerebral hemorrhage, periprocedural or unspecified hemorrhage, or need for blood transfusion), and the utilization of invasive management procedures such as coronary angiography, percutaneous coronary intervention, and coronary artery bypass grafting.

### 2.4. Statistical Analysis

Statistical analysis was conducted using IBM SPSS version 29. Due to skewed data, continuous variables were presented as median and an interquartile range, while categorical data were reported as frequencies and percentages. Pearson’s chi-square test was used for categorical variable comparisons, and ANOVA was employed for continuous variables. AHRQ-specified sampling weights were applied to estimate the total discharges. To assess the relationship between in-hospital outcomes and CKM stages, multivariable logistic regression models were utilized. Results were expressed as odds ratios (ORs) with 95% confidence intervals (Cis). All models were adjusted for baseline differences between the groups, controlling for the following covariates: age, gender, race, weekend admission, hospital bed size, region and location/teaching status, CABG, PCI, CA, ventricular fibrillation (VF), ventricular tachycardia (VT), valvular heart disease, smoking status, chronic liver disease, chronic lung disease, dementia, smoking anemia, thrombocytopenia, coagulopathies, and malignancies.

## 3. Results

In this study, a total of 2,959,244 cases of AMI were identified. After excluding cases with missing data, the final study cohort consisted of 2,768,154 cases (93.5%). The study cohort was stratified into groups based on CKM stage: CKM Stage 0 included 68,685 cases (2.5%), Stage 1 included 33,115 cases (1.2%), Stages 2 and 3 included 255,620 cases (9.2%), and Stage 4 included 2,410,735 cases (87.1%). Stage 4 was further divided into subgroup 4A with 1,695,450 cases (61.2%) and subgroup 4B with 715,285 cases (25.8%).

The baseline characteristics of patients with AMI categorized by CKM stages are shown in Table 1. Mean age increased significantly with advancing CKM stages, ranging from 56.7 years in Stage 1 to 73.2 years in Stage 4b (*p*-value < 0.001). The proportion of females decreased from 52% in CKM Stage 1 to 39.4% in Stage 4A and 40.6% in Stage 4B (*p* < 0.001). As CKM stages advanced, there was an increase in the prevalence of comorbidities, including valvular heart disease (from 4.9% in Stage 0 to 20.9% in Stage 4B, *p* < 0.001). The prevalence of dementia and anemia was significantly higher in Stage 4B (10.4% and 48.8%, respectively) compared to Stage 0 (6.8% and 22.2%, respectively, *p* < 0.001). A general decline was observed in the prevalence of intracerebral hemorrhage (from 1.8% in Stage 0 to 0.5% in Stage 4B), solid malignancy (from 7.3% to 3.3%), and metastatic malignancy (from 5.4% to 1.3%), with the most significant reduction in CKM Stage 4 (*p* < 0.001). No clear pattern emerged in the prevalence of smoking, chronic liver disease, hematologic malignancy, or thrombocytopenia across CKM stages. Additional baseline demographic and clinical characteristics are presented in Table 1.

### 3.1. In-Hospital Procedures and Outcomes

#### Crude Rates

**Figure 2:**
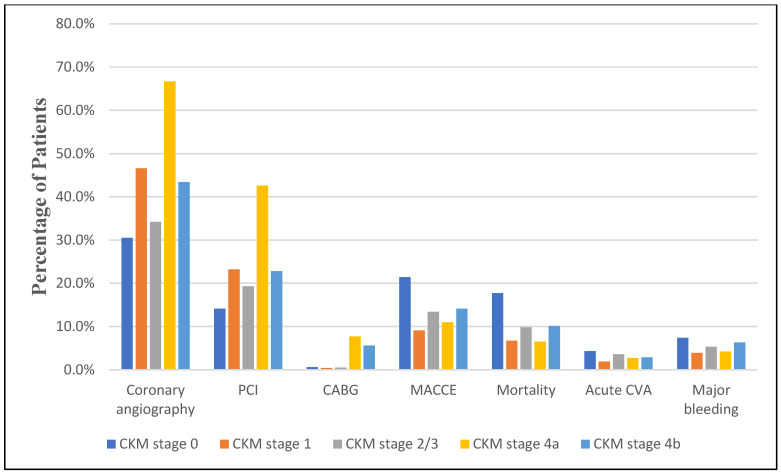
In-hospital procedures and outcomes of patients with MI based on CKM stages.

Table 2 and Figure 2 demonstrate the relationship between in-hospital procedures and outcomes across varying CKM stages. We observed an increasing performance of invasive cardiac procedures across CKM stages. The prevalence of coronary angiography (CA) increased from 30.5% in Stage 0 to 66.7% in Stage 4A and 43.4% in Stage 4B (*p* < 0.001). Similarly, the rates of percutaneous coronary intervention (PCI) rose from 14.1% in Stage 0 to 42.6% in Stage 4a and 22.8% in Stage 4b (*p* < 0.001). Coronary artery bypass grafting (CABG) procedures also showed an upward trend, increasing from 0.6% in Stage 0 to 7.7% in Stage 4A and 5.6% in Stage 4B (*p* < 0.001).

Significant differences in in-hospital outcomes were observed across CKM stages. The incidence of major adverse cardiovascular and cerebrovascular events (MACCEs) decreased from 21.4% in Stage 0 to 11% in Stage 4A and 14.1% in Stage 4B (*p* < 0.001). In-hospital mortality rates showed a similar trend, declining from 17.7% in Stage 0 to 6.5% in Stage 4A and 10.1% in Stage 4B (*p* < 0.001). The prevalence of acute cerebrovascular accidents (CVAs) and major bleeding events also decreased across CKM stages, with the lowest rates identified in CKM Stage 1 (*p* < 0.001 for both outcomes).

The mean length of stay (LOS) increased from 5.7 days in Stage 0 to 7.0 days in Stage 4B (*p* < 0.001). Mean total charges followed a similar pattern, from USD 89,890 in Stage 0 to USD 71,472 in Stage 1 to USD 96,495 in Stage 4A and USD 101,843 in Stage 4B (*p* < 0.001).

**Figure 3:**
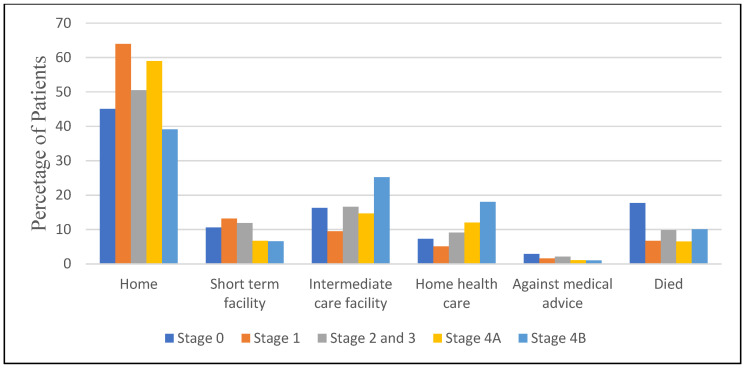
Disposition of patients based on CKM stages.

Figure 3 represents the disposition of patients with AMI across different stages of CKM. The majority of patients across all stages were discharged home, with the highest percentage observed in Stage 1 (63.9%) and the lowest in Stage 4B (39.1%). Interestingly, the need for intermediate care facilities and home healthcare increased with disease progression, peaking at 25.2% and 18%, respectively, for Stage 4B. Short-term facility utilization was highest in Stage 1 (13.2%) and lowest in Stage 4B (6.6%). The percentage of patients leaving against medical advice was relatively low across all stages, with a maximum of 2.9% in Stage 0.

### 3.2. Adjusted Analysis

Table 3 presents the adjusted odds ratios (ORs) for in-hospital procedures and outcomes stratified by CKM stages. Regarding in-hospital procedures, the odds of undergoing coronary angiography, PCI, and CABG generally increased with advancing CKM stages. Coronary angiography showed the strongest association in CKM Stage 4A (aOR: 6.86, 95% CI: 6.73–6.99, *p*-value < 0.001) and Stage 4B (aOR: 3.87, 95% CI: 3.80–3.95, *p*-value < 0.001). Similarly, the likelihood of PCI was highest in Stage 4A (aOR: 5.93, 95% CI: 5.79–6.08, *p*-value < 0.001) and Stage 4B (aOR: 4.14, 95% CI: 4.04–4.24, *p*-value < 0.001). CABG also demonstrated an increase in odds for advanced CKM stages, with Stage 4A showing an exceptionally high aOR of 20.70 (95% CI: 18.71–22.91, *p*-value < 0.001).

In terms of clinical outcomes, an unexpected trend emerged. The odds of mortality decreased with advancing CKM stages, with the lowest risk in Stage 4A (aOR: 0.25, 95% CI: 0.25–0.26, *p*-value < 0.001). MACCEs showed consistently lower odds across all CKM stages compared to Stage 0, with the lowest in Stage 4A (aOR: 0.44, 95% CI: 0.43–0.45, *p*-value < 0.001). Acute CVA did not demonstrate a clear linear relationship with CKM severity, with the lowest odds in Stage 1 (aOR: 0.56, 95% CI: 0.52–0.62, *p*-value < 0.001). Major bleeding events also showed lower odds across all CKM stages, with the lowest in Stage 4B (aOR: 0.61, 95% CI: 0.59–0.63, *p*-value < 0.001).

## 4. Discussion

In this study, we analyzed data from 2,768,154 patients with MI, stratified by pre-existing CKM stages, between 2016 and 2019. Our results showed several interesting findings. First, a significant increase in mean age and male prevalence was observed as CKM severity advanced. Second, our analysis revealed a complex association between CKM syndrome stages, in-patient management, and outcomes among patients with AMI. Patients with severe CKM were more likely to undergo in-hospital procedures, including coronary angiography, percutaneous coronary intervention, and coronary artery bypass grafting. Finally, the study revealed an unexpected relationship between CKM severity and patient outcomes. Contrary to what might be expected, patients without CKM risk factors (Stage 0) demonstrated higher rates of adverse outcomes compared to other stages. Among Stages 1–4B, there was no consistent graded association between CKM stage and outcomes, with Stage 4A often showing better outcomes than earlier stages.

Our analysis revealed that among the study population of patients with MI, the largest proportion of patients was classified as having CKM Stage 4, with a notable trend of increasing age in more advanced CKM stages. This observation aligns with prior studies, demonstrating that advancing age is associated with an increased prevalence of comorbidities such as diabetes, obesity, and cardiovascular diseases [5,6]. The progression of metabolic and cardiovascular diseases with age likely contributes to this trend, as these conditions are key components of CKM syndrome.

We reported that MI patients in severe CKM stages are more likely to undergo invasive cardiac procedures, including PCI, CABG, and coronary angiography. This finding presents an interesting contrast to some existing literature. For instance, Abramov et al. found that patients with pre-existing heart failure, a common feature in advanced CKM stages, were less likely to receive invasive management (coronary angiography and PCI) for acute myocardial infarction [7]. In contrast, Bucholz et al. [8] investigated Medicare beneficiaries hospitalized with AMI over a 17-year follow-up period to evaluate the association between higher BMI and survival outcomes after AMI. They observed that overweight and obese patients had the highest rates of undergoing PCI or CABG within the first 30 days following AMI [8]. Similarly, Keller et al. [9] reported that the likelihood of obese MI patients undergoing PCI was 58.5%, compared to 47.9% in non-obese MI patients. A similar trend was observed for CABG and coronary angiography, with higher rates of these procedures among obese patients with MI compared to their non-obese counterparts [9]. Despite patients with severe CKM being at greater risk, they are more likely to undergo invasive cardiac procedures. This may be due to their more severe health conditions, where urgent and aggressive medical intervention is often required to improve outcomes.

The unexpected relationship between advanced CKM stages and better outcomes may be attributed to several factors. Patients with severe CKM likely receive earlier and more aggressive intervention due to their higher risk profile, as well as more intensive medical therapy and closer monitoring. Additionally, patients who reach advanced CKM stages may represent a subset of individuals more resilient to adverse outcomes. Interestingly, patients without CKM risk factors (Stage 0) demonstrated worse outcomes compared to other stages, suggesting a complex interplay of factors influencing patient prognosis. This phenomenon of better outcomes in higher-risk groups aligns with observations in other conditions, such as the “obesity paradox” in cardiovascular disease [9].

Our study revealed that patients classified as CKM Stage 0 who experienced AMI demonstrated higher mortality rates compared to those in more advanced CKM stages. This observation suggests that patients presenting with AMI in the absence of traditional cardiovascular risk factors may represent a distinct population from those experiencing AMI in the context of established cardiovascular comorbidities. This finding aligns with the results of Figtree et al., who reported higher mortality rates in STEMI patients without standard modifiable risk factors, particularly in women [10]. The CKM Stage 0 cohort in our study exhibited higher rates of malignancy and coagulopathy, indicating that AMI in this group might stem from non-standard etiologies. This finding aligns with previous research demonstrating poorer outcomes in patients with AMI and concomitant cancer. Bharadwaj et al. [11] revealed that cancer patients experiencing AMI had higher in-hospital mortality rates and were less likely to receive guideline-recommended AMI treatments compared to non-cancer patients [11]. Similarly, a large-scale study by Kobo et al. [12] analyzing 20.6 million emergency department records in the USA found that cardiovascular disease encounters in cancer patients were associated with higher mortality rates [12]. Another relevant study by Sedhom et al. [13] focused on AMI outcomes in patients with hypercoagulable conditions. Their research revealed that these patients had lower utilization of invasive evaluation procedures and higher mortality rates in the setting of AMI [13]. Furthermore, our observations are consistent with recent studies examining the non-cardiac drivers of AMI. For instance, a recent analysis by Sedhom et al. [14] investigated AMI outcomes in patients with burns. Their findings showed significantly higher mortality rates among burn patients who experienced AMI compared to those without burns, highlighting the complex interplay between systemic inflammation, hypercoagulability, and cardiovascular outcomes [14]. These findings collectively suggest that patients presenting with AMI in the absence of traditional cardiovascular risk factors may require special consideration in terms of diagnosis and management. The lower rates of invasive procedures observed in our CKM Stage 0 group, combined with higher mortality, indicate a potential gap in current AMI management strategies for this unique population.

While this study provides meaningful insights into the impact of CKM syndrome on the outcomes of patients with MI, it is important to acknowledge several limitations. The study relies on data from an administrative dataset, and the use of ICD-10 codes may lead to misclassification, incomplete diagnoses, and missing procedures. Although patients coded as having type 2 AMI were excluded, it is possible that some patients in the current analysis had uncoded type 2 AMI. Additionally, the NIS database does not capture multiple hospitalizations of the same patient within a year or across different years. Since it focuses on hospital stays, it is possible for the same patient to be counted more than once if admitted more than once during the study period, leading to potential overestimation or duplication of certain events in the analysis. The NIS database provides data from various hospitals, which may lead to inconsistencies in treatment approaches and patient classification due to differing protocols at each facility. The NIS also lacks information on the quality of care. Additionally, this study focused on in-hospital outcomes due to the limitations of the NIS database, which does not provide post-discharge follow-up data. While this approach allows for a comprehensive analysis of immediate post-MI care and short-term outcomes, it may not capture longer-term events or complications that could potentially alter the observed relationship between CKM stages and outcomes. Furthermore, the CKM classification depends on ICD codes, which may not fully reflect the syndrome’s complexity. It is worth noting that while we used BMI codes in addition to ’obesity’, other body measures may be more appropriate to capture obesity with metabolic consequences [15]. Lastly, the NIS database lacks detailed information on key factors like lifestyle, diet, and physical activity, which can influence CKM risk factors. The apparent reduction in adverse events in advanced CKM stages could be partially explained by the lack of information on quality of care. Other possible explanations include increased clinical vigilance for high-risk patients, more intensive medical management, potential survival bias where patients reaching advanced CKM stages may be inherently more resilient, and the possibility that advanced CKM patients receive care at more experienced centers. Additional studies are needed to evaluate the optimal diagnosis and management of AMI in patients without traditional cardiovascular risk factors. Future research should focus on identifying specific mechanisms underlying AMI in this population, developing targeted strategies to improve their outcomes, and better understanding the interplay between CKM severity, treatment strategies, and long-term prognosis.

## 5. Conclusions

This study highlights the complex relationship between CKM syndrome and the management and prognosis of patients with MI. Our findings reveal an increase in the use of coronary angiography, PCI, and CABG in the more severe stages of CKM. Notably, patients with CKM Stage 0 demonstrated higher rates of adverse outcomes compared to other stages. Among Stages 1–4B, there was no consistently graded association between the CKM stage and outcomes. Patients with CKM Stage 0 who experience AMI in the absence of traditional cardiovascular risk may represent a unique population with less frequent utilization of invasive evaluation and higher mortality compared to patients with CKM syndrome, and this group deserves further evaluation.

## Figures and Tables

**Table 1:** Demographics, hospital record characteristics, and comorbidities of patients with MI based on CKM stages.

	CKM Stage					
	0	1	2 and 3	4A	4B	*p*-Value
**NIS discharge weight**	68,680	33,095	255,570	1,695,070	715,145	
**Mean age**	58.9	56.7	66.2	67.2	73.1	<0.001
**Female, %**	45.3%	52%	50.30%	39.4%	40.6%	<0.001
**Ethnicity**						<0.001
White	75.1%	70%	67.6%	77.3%	69%	
Black	11.4%	16.1%	16.6%	10.2%	16.5%	
Hispanic	7.9%	9%	9.5%	7%	8.3%	
Asian	2.3%	1.7%	3.1%	2.2%	3.2%	
Native	0.6%	0.4%	0.5%	0.5%	0.5%	
Other	2.8%	2.80%	2.90%	2.80%	2.5%	
**Hospital region**						<0.001
Northeast	24.1%	22.2%	22.4%	20.5%	20.6%	
Midwest or North Central	20.6%	23.9%	22%	24.5%	24.6%	
South	39%	37%	39.7%	40.6%	39.9%	
West	16.3%	16.80%	15.90%	14.4%	14.9%	
**Hospital bed size**						<0.001
Small	19.5%	20.5%	20.7%	16.6%	17.6%	
Medium	29.4%	30%	31.2%	29.8%	30.1%	
Large	51.1%	49.5%	48%	53.7%	52.3%	
**Hospital location/teaching status**						<0.001
Rural	10.3%	8.4%	8%	8.3%	8.3%	
Urban non-teaching	25.6%	25.4%	23.9%	23.5%	23.5%	
Teaching	64.1%	66.1%	68.1%	68.2%	68.2%	
**Median ZIP income**						<0.001
1st quartile	31.9%	30.8%	33.2%	30.5%	32.4%	
2nd quartile	27.6%	26.7%	26.9%	27.8%	27.2%	
3rd quartile	22.4%	24.4%	22.3%	23.5%	23%	
4th quartile	18%	18.2%	17.5%	18.3%	17.3%	
**Primary expected payer**						<0.001
Medicare	40.4%	33%	56%	57.6%	79.5%	
Medicaid	18.1%	15.9%	12.2%	9.6%	6.4%	
Private insurance	29.5%	41.1%	24%	25.1%	10.6%	
Self-pay	7.9%	6.4%	4.7%	4.6%	1.5%	
No charge	0.6%	0.7%	0.5%	0.4%	0.1%	
Other	3.5%	2.9%	2.%	2.7%	1.9%	
**Record characteristics**						
STEMI	21.6%	18.2%	17.9%	26.4%	11.7%	<0.001
NSTEMI	78.4%	81.8%	82.1%	73.6%	88.3%	<0.001
Cardiac arrest	7.80%	3.9%	4.4%	3.6%	4.1%	<0.001
Ventricular fibrillation	3.70%	2.4%	1.7%	3.3%	1.9%	<0.001
Ventricular tachycardia	4.50%	4.1%	3.3%	7%	6.7%	<0.001
**Comorbidities**						
Valvular heart disease	4.9%	5%	6.9%	13.4%	20.9%	<0.001
Smoking	39.7%	46.3%	40.8%	49.9%	39.4%	<0.001
Dementia	6.8%	2.5%	10.2%	6.5%	10.4%	<0.001
Anemia	22.2%	16.3%	23.8%	21.1%	48.8%	<0.001
Thrombocytopenia	8.3%	5.5%	6.2%	5.7%	9.1%	<0.001
Coagulopathy	6.2%	3.2%	3.0%	2.1%	3.0%	<0.001
Chronic liver disease	1.1%	0.8%	1.1%	0.7%	1.2%	<0.001
Intracerebral hemorrhage	1.8%	1.1%	1.2%	0.6%	0.5%	<0.001
Hematologic malignancy	2.0%	1.2%	1.6%	1.2%	1.9%	<0.001
Solid malignancy	7.3%	3.3%	4.9%	3.0%	3.3%	<0.001
Metastatic malignancy	5.4%	2%	3%	1.4%	1.3%	<0.001

**Table 2:** In-hospital procedures and outcomes of patients with MI based on CKM stages.

	CKM Stage					
	0	1	2 and 3	4A	4B	*p*-Value
**NIS discharge weight**	68,680	33,095	255,570	1,695,070	715,145	
**In-Hospital Procedures**						
Coronary Angiography	30.5%	46.6%	34.2%	66.7%	43.4%	<0.001
PCI	14.1%	23.2%	19.3%	42.6%	22.8%	<0.001
CABG	0.6%	0%	0.5%	7.7%	5.6%	<0.001
Thrombolysis	0.1%	0.1%	0.1%	0.1%	0.1%	<0.001
Mechanical Ventilation	22.7%	11.7%	11.9%	9.8%	12.2%	<0.001
Circulatory support (inc. IABP, LV assist device and ECMO).	1.8%	0.9%	1.0%	4.6%	3.6%	<0.001
**In-Hospital Outcomes**						
MACCE	21.4%	9.10%	13.4%	11%	14.1%	<0.001
Mortality	17.7%	6.7%	9.80%	6.50%	10.1%	<0.001
Acute CVA	4.3%	1.9%	4%	2.7%	2.9%	<0.001
Major Bleeding	7.4%	3.9%	5.3%	4.2%	6.3%	<0.001
**Length of stay, days, mean**	5.72	4.05	4.6	5.06	7	<0.001
**Total charge, USD, mean**	USD 89,890.11	USD 71,472.06	USD 70,846.81	USD 96,495.32	USD 101,842.68	<0.001

**Table 3:** Multivariable analysis presenting ORs for in-hospital procedures and outcomes stratified by CKM stages.

	CKM Stage 1		CKM Stage 2 and 3		CKM Stage 4A		CKM Stage 4B	
	OR (95% CI)	*p*-Value	OR (95% CI)	*p*-Value	OR (95% CI)	*p*-Value	OR (95% CI)	*p*-Value
**CA**	1.97 (1.91–2.02)	<0.001	1.68 (1.64–1.71)	<0.001	6.86 (6.73–6.99)	<0.001	3.87 (3.80–3.95)	<0.001
**PCI**	1.90 (1.83–1.97)	<0.001	1.97 (1.91–2.02)	<0.001	5.93 (5.79–6.08)	<0.001	4.14 (4.04–4.24)	<0.001
**CABG**	0.76 (0.62–0.93)	0.009	1.07 (0.96–1.21)	0.223	20.70 (18.71–22.91)	<0.001	8.49 (7.67–9.40)	<0.001
**MACCE**	0.47 (0.45–0.49)	<0.001	0.58 (0.56–0.59)	<0.001	0.44 (0.43–0.45)	<0.001	0.50 (0.49–0.52)	<0.001
**Mortality**	0.45 (0.43–0.48)	<0.001	0.46 (0.44–0.47)	<0.001	0.25 (0.25–0.26)	<0.001	0.34 (0.33–0.35)	<0.001
**Acute CVA**	0.56 (0.52–0.62)	<0.001	0.94 (0.90–0.98)	0.007	1.05 (1.01–1.09)	0.024	0.83 (0.80–0.87)	<0.001
**Major bleeding**	0.69 (0.64–0.73)	<0.001	0.74 (0.71–0.76)	<0.001	0.75 (0.73–0.78)	<0.001	0.61 (0.59–0.63)	<0.001

Reference: CKM STAGE 0; adjusted for age, gender, weekend admission, hospital bed size, region and location/teaching status, STEMI, cardiac arrest, cardiogenic shock, VF, VT, valvular heart disease, chronic liver disease, chronic lung disease, anemia, thrombocytopenia, coagulopathies, malignancies, coronary angiography (CA), PCI, and CABG.

## Data Availability

The data presented in this study are available upon request from the corresponding author. The data are not publicly available due to privacy or ethical restrictions.

## Supplementary Materials

The following supporting information can be downloaded at: https://www.mdpi.com/article/10.3390/diseases13040097/s1. Table S1: ICD-10 codes for patient characteristics, in-hospital procedures, and post-procedural complications; Table S2: Definition of CKM syndrome staging according to the American Heart Association; Table S3: Modified CKM Syndrome Staging for Study Analysis based on ICD-10 Codes.
