# Supplementary material for: Impact of Cardiovascular–Kidney–Metabolic Syndrome Staging on Myocardial Infarction Outcomes: A Retrospective Analysis of 2.7 Million Patients
Source: Diseases. 2025 Mar 27;13(4):97. doi: 10.3390/diseases13040097 (PMC12026123; doi:10.3390/diseases13040097)
Supplement: Supplementary file 1 [file diseases-13-00097-s001.zip › diseases-3483382-supplementary.pdf]

Table S1: : ICD-10 codes for patient characteristics, in hospital procedures and post-procedural complications

Variable	Codes
**Patient and Record Characteristics**	
Ischemic Heart disease	I25.2;I25.5; Z95.1; I25.7x.; Z98.61; Z95.5
Cerebrovascular disease	I65-69
Renal Disease	I70.1; I15.0 ; N18.x (excluded Nephrotic synd<br>N04.x and chronic renal<br>calculous whydronephrosis<br>N13.2)
Aortic Disease	I70.0
Peripheral Vascular Disease of extremities	I70.2x-I70.9x; I73.x; Z98.62
AMI	I21
STEMI	I21.0-3
Cardiac Arrest	I46.x
Ventricular Fibrillation	I49.01
Ventricular tachycardia	I47.2
Cardiogenic Shock	R57.0
Heart failure	I50
Valvular disease	I05-08; I34-37
Atrial fibrillation/flutter	I48
Hypertension	I10-16
Dyslipidaemia	E78
Diabetes	E08-13
Smoking	Z87.891 , Z72.0
Obesity	E66.9
Pre-diabetes	R73.03
Anaemia	D55-59
Thrombocytopenia	D69.3-.6
Coagulopathy	D65-68;D69.0-.2
Dementia	F01-03
Chronic Liver Disease	K73-74
Homelessness	Z59.0
Solid malignancy	C00.x-C76.x; C80.x
Hematologic Malignancies	C81-96
Metastatic cancer	C77.x-79.x
**In Hospital Procedures**	
Coronary Angiography	B211x
PCI	02703x/13x/23x/33x
CABG	02100*/04*/10*/14*/20*/24*/30*/34*
Thrombolysis	3E07317
Mechanical Ventilation	5A19054/35Z/45Z/55Z
Circulatory support (inc. IABP, LV assist device and ECMO).	5A02x, 5A1522G, 5A15A2G, 5A15A2H
**In Hospital Outcomes**	
Acute Ischemic CVA	I63

Coronary artery dissection	I2542
Pericardial effusion (incl tamponade)	I23.0 I31.2 I31.4 I31.3
Tamponade	I31.4
Dressler‘s syndrome	I24.1
Post MI angina	I23.7
Intracardiac Thrombus	I23.6
Mechanical complications	I23.1-I23.5
GI bleed	K92.0-92.2; K25.0-25.2; K25.4-25.6; K26.0-26.2; K27.0-27.2; K27.4-27.6; K28.0-28.2; K28.4-28.6
Retroperitoneal Bleed	K66.1
Intracranial Haemorrhage	I60-62

Table S2: Definition of CKM syndrome staging according to the American Heart Association (2)

CKM syndrome stages	Definition
**Stage 0: No CKM risk factors**	Individuals with normal BMI and waist circumference, normoglycemia, normotension, a normal lipid profile, and no evidence of CKD or subclinical or clinical CVD
**Stage 1: Excess or dysfunctional adiposity**	Individuals with overweight/obesity, abdominal obesity, or dysfunctional adipose tissue, without the presence of other metabolic risk factors or CKD<br>BMI ≥25 kg/m^2 (or ≥23 kg/m^2 if Asian ancestry),<br>Waist circumference ≥88/102 cm in women/men (or if Asian ancestry ≥80/90 cm in women/men), or<br>Fasting blood glucose ≥100–124 mg/dL or HbA1c between 5.7% and 6.4%
**Stage 2: Metabolic risk factors and CKD**	Individuals with metabolic risk factors (hypertriglyceridemia [≥135 mg/dL], hypertension, MetS,† diabetes), or CKD
**Stage 3: Subclinical CVD in CKM**	Subclinical ASCVD or subclinical HF among individuals with excess/dysfunctional adiposity, other metabolic risk factors, or CKD<br>Subclinical ASCVD to be principally diagnosed by coronary artery calcification (subclinical atherosclerosis by coronary catheterization/CT angiography also meets criteria)<br>Subclinical HF diagnosed by elevated cardiac biomarkers (NT-proBNP ≥125 pg/mL, hs-troponin T ≥14 ng/L for women and ≥22 ng/L for men, hs-troponin I ≥10 ng/L for women and ≥12 ng/L for men) or by echocardiographic parameters, with a combination of the 2 indicating highest HF risk.<br>Risk equivalents of subclinical CVD<br>Very high-risk CKD (stage G4 or G5 CKD or very high risk per KDIGO classification)<br>High predicted 10-y CVD risk
**Stage 4: Clinical CVD in CKM**	Clinical CVD (coronary heart disease, HF, stroke, peripheral artery disease, atrial fibrillation) among individuals with excess/dysfunctional adiposity, other CKM risk factors, or CKD<br>Stage 4a: no kidney failure<br>Stage 4b: kidney failure present

Legend: ASCVD indicates atherosclerotic cardiovascular disease; BMI, body mass index; CKD, chronic kidney disease; CKM, cardiovascular-kidney-metabolic; CT, computed tomography; CVD, cardiovascular disease; HbA1c, hemoglobin A1c; HDL, high-density lipoprotein; HF, heart failure; hs-troponin, high-sensitivity troponin; KDIGO, Kidney Disease Improving Global Outcomes; MetS, metabolic syndrome; and NT-proBNP; N-terminal pro-B-type natriuretic peptide.

† MetS is defined by the presence of 3 or more of the following: (1) waist circumference ≥88 cm for women and ≥102 cm for men (≥80 cm for women and ≥90 cm for men if Asian ancestry); (2) HDL cholesterol <40 mg/dL for men and <50 mg/dL for women; (3) triglycerides ≥150 mg/dL; (4) elevated blood pressure (systolic blood pressure ≥130 mm Hg or diastolic blood pressure ≥80 mm Hg and/or use of antihypertensive medications); and (5) fasting blood glucose ≥100 mg/dL.

Table S3: Modified CKM Syndrome Staging for Study Analysis based on ICD-10 Codes

CKM syndrome stages	Definition
**Stage 0: No CKM risk factors**	None of the ICD-10 codes below are present
**Stage 1: Excess or dysfunctional adiposity**	E66.9 (Obesity, unspecified) or R73.03 (Prediabetes)<br>No codes listed in advanced stages
**Stages 2 and 3 (combined): Metabolic risk factors and conditions**	At least one of:<br>E08-13 (Diabetes)<br>N18.9 (Chronic kidney disease)<br>I10-16 (Hypertension)<br>E78 (Dyslipidaemia)
**Stage 4: Clinical CVD in CKM**	At least one of:<br>I25.2;I25.5; Z95.1; I25.7x.; Z98.61; Z95.5 (Ischemic Heart Disease)<br>I65-69 (CVD)<br>I70.2x-I70.9x; I73.x; Z98.62 (PVD)<br>E08-13 (Diabetes)<br>N18.9 (Chronic kidney disease)<br>I10-16 (Hypertension)<br>E78 (Dyslipidaemia)<br>I48 (Atrial fibrillation/flutter)<br>I50 (Heart Failure)<br>Stage 4a: no kidney failure<br>Stage 4b: kidney failure present N18.9 (Chronic kidney disease)
